# Down-regulation of miR-214 reverses erlotinib resistance in non-small-cell lung cancer through up-regulating LHX6 expression

**DOI:** 10.1038/s41598-017-00901-6

**Published:** 2017-04-10

**Authors:** Jinrong Liao, Jinghui Lin, Dong Lin, Changyan Zou, Jessica Kurata, Renjang Lin, Zhiyong He, Ying Su

**Affiliations:** 1 Department of Radiobiology, Fujian Cancer Hospital, Fujian Medical University Cancer Hospital, Fuzhou, 350014 Fujian Province P.R. China; 2 Department of Thoracic Medical Oncology, Fujian Cancer Hospital, Fujian Medical University Cancer Hospital, Fuzhou, 350014 Fujian Province P.R. China; 3 Department of Molecular and Cellular Biology, Irell & Manella Graduate School of Biological Sciences, Beckman Research Institute of the City of Hope, Duarte, CA 91010 USA

## Abstract

Epidermal growth factor receptor (EGFR) tyrosine kinase inhibitors (TKIs) are standard treatments for advanced non-small-cell lung cancer (NSCLC) patients. However, acquired resistance to EGFR-TKIs is widely detected across the world, and the exact mechanisms have not been fully demonstrated until now. This study aimed to examine the role of miR-214 in the acquired resistance to erlotinib in NSCLC, and elucidate the underlying mechanisms. qRT-PCR assay detected higher miR-214 expression in the plasma of NSCLC patients with acquired EGFR-TKI resistance than prior to EGFR-TKI therapy, and in the generated erlotinib-resistant HCC827 (HCC827/ER) cells than in HCC827 cells. Bioinformatics analysis and dual-luciferase reporter assay indentified LHX6 as a direct target gene of miR-214, and LHX6 expression was detected to be down-regulated in erlotinib-resistant HCC827 cells. Transwell invasion assay revealed that overexpressing LHX6 reversed the increase in the invasive ability of HCC827 cells induced by miR-214 overexpression, and the CRISPR-Cas9 system-mediated LHX6 knockdown reversed the reduction in the invasion of erlotinib-resistant HCC827 cells caused by miR-214 down-regulation. The results of the present study demonstrate that down-regulation of miR-214 may reverse acquired resistance to erlotinib in NSCLC through mediating its direct target gene LHX6 expression.

## Introduction

Lung cancer is the most common tumor and the leading cause of cancer-related mortality worldwide^1^. Non-small-cell lung cancer (NSCLC), which accounts for 80% to 85% of all lung cancer patients, is estimated to have an only 15% 5-year survival and characterized by a high recurrence^2^.

Currently, epidermal growth factor receptor (EGFR) mutation is the most common type of gene mutations detected in Asian populations with lung cancer, which mainly includes exon 21 L858R point mutation and exon 19 deletion (19 del) mutation^3^. EGFR is also identified as the therapeutic target of EGFR tyrosine kinase inhibitors (TKIs), a new class of targeted therapeutic agents against lung cancer^4^. First-generation EGFR-TKIs have become the standard treatment for advanced NSCLC with EGFR mutations^5–7^. It is reported that EGFR-TKIs, such as erlotinib and gefitinib, achieve a median progression-free survival (PFS) of 8 to 16 months in the treatment of EGFR-mutant NSCLC patients, and then acquired drug resistance may develop^8^. There are some hypotheses proposed to explain the mechanisms of acquired EGFR-TKI resistance^9–11^; however, the exact mechanisms have not been fully elucidated.

microRNA (miRNA), a class of small non-coding RNA molecules containing 19 to 23 nucleotides that serve as key mediators in post-transcriptional gene regulation, has been found to be involved in the acquired resistance to EGFR-TKIs in NSCLC^12–14^. It has been shown that miRNA affects the biological behaviors of multiple cancers^15–17^. miR-214, a vertebrate-specific family of miRNA precursor^18^, has shown diagnostic and prognostic values in gastric cancer^19^, pancreatic cancer^20^, cervical cancer^21^, breast cancer^22^, hepatocellular carcinoma^23^, ovarian cancer^24^, melanoma^25^ and Sézary syndrome^26^. In addition, it has been reported that miR-214 contributes to the resistance to chemotherapeutics in cancers^27, 28^. In human ovarian cancer cells, miR-214 was found to induce cisplatin resistance primarily through targeting the PTEN/Akt pathway^29^, and in human lung cancer cell line HCC827, miR-214 was reported to regulate the acquired resistance to gefitinib via PTEN/AKT signaling pathway^30^. Taking previous reports together, miR-214 may be involved in the proliferation, cell cycle and apoptosis of cancer cells through directly mediating target PTEN, NECL2, FGFR, NRAS, beta-catenin, UBC9, EZH2 and P53 genes or indirectly regulating downstream signaling molecules^27, 28^. It is therefore hypothesized that miR-214 may mediate the resistance of EGFR-mutant NSCLC to TKIs through mediating cancer cell apoptosis-associated target genes. To test this hypothesis, this study was designed to examine the role of miR-214 in the acquired resistance to erlotinib in NSCLC, and elucidate the underlying mechanisms.

## Results

### MiR-214 expression is up-regulated in NSCLC with acquired EGFR-TKI resistance

**Figure 1:**
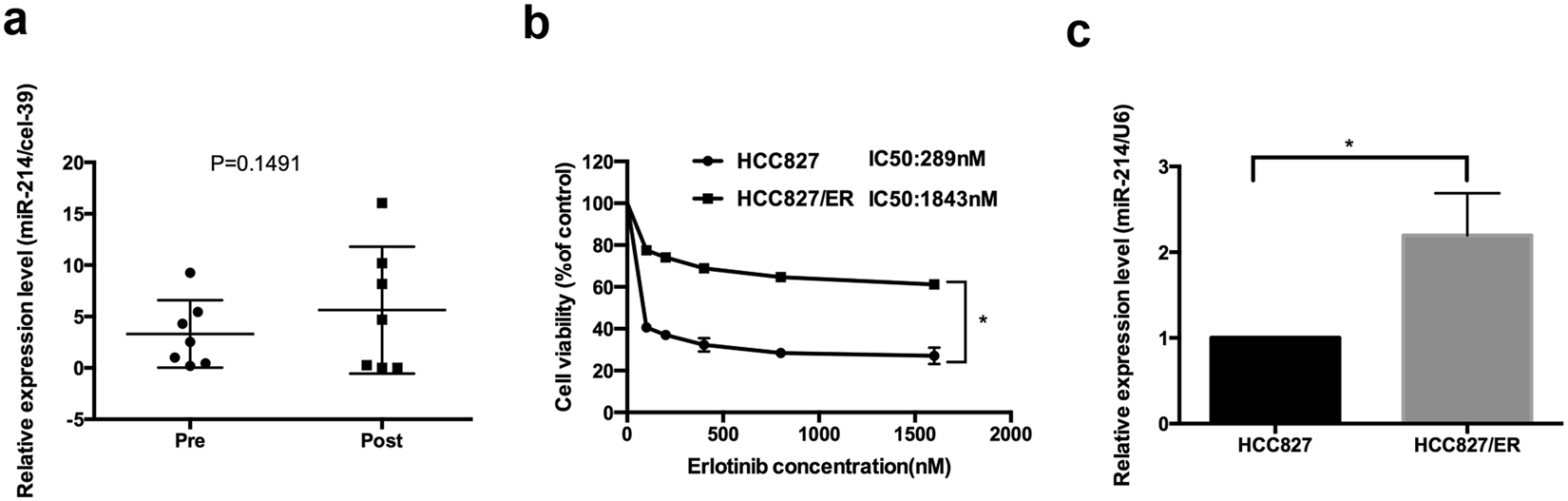
miR-214 expression in HCC827 and HCC827/ER cells and in the plasma of NSCLC patients pre- and post-treatment with EGFR-TKIs. (**a**) qRT-PCR assay revealed an increase in the miR-214 expression in the plasma of NSCLC patients with acquired EGFR-TKI resistance than in NSCLC patients prior of EGFR-TKI therapy (*P* = 0.398). (**b**) MTS assay showed a 289 nM IC_50_ for HCC827 cells and a 1,843 nM erlotinib IC_50_ for HCC827/ER cells. *P < 0.05; (**c**) qRT-PCR assay showed significant up-regulation of miR-214 expression in HCC827/ER cells than in HCC827 cells. *P < 0.05; All data are expressed as mean ± SD from three independent experiments.

First, we compared blood samples from patients who developed resistance to erlotinib for change of plasma miR-214. Of the seven NSCLC patients harboring EGFR mutation enrolled in this study, who developed disease progression and had acquired EGFR-TKI resistance, the plasma was sampled for quantifying miR-214 expression. qRT-PCR assay showed higher relative miR-214 expression level in the plasma of NSCLC patients with acquired EGFR-TKI resistance than prior to EGFR-TKI therapy (5.63 ± 2.33 vs. 3.31 ± 1.24, *P* = 0.398) (Fig. 1a). The change was not statistically significant probably due to the small patient numbers or that the plasma miRNA-214 level might not strictly correlate with the tumor miR-214 level.

To examine the role of miR-214 expression in acquired resistance to EGFR-TKI in NSCLC, we generated an EGFR-TKI resistant cell line. HCC827 cells were exposed to erlotinib by dose escalation from 10 to 1,600 nM for 6 months, and then cells were incubated in erlotinib-free RPMI 1640 medium for further 2 months. MTS assay showed a 1,843 nM erlotinib IC_50_ for HCC827 cells that were subject to drug treatment and a 289 nM IC_50_ for untreated HCC827 cells (Fig. 1b), indicating the successful development of erlotinib-resistant cells (HCC827/ER cells). qRT-PCR assay revealed a significant increase in miR-214 expression in HCC827/ER cells than in HCC827 cells (Fig. 1c). Our findings demonstrate that the elevated miR-214 expression may correlate with the acquired EGFR-TKI resistance in both HCC827 cells and NSCLC patients.

### LHX6 is a direct target gene of miR-214

LHX6 was reported to suppress the growth and invasion of breast cancer cells through inhibiting Wnt/β‑catenin signaling pathway^31^, and has been putatively identified as a tumor suppressor gene in lung cancer through regulating apoptosis-related genes p53 and Bcl-2, cell cycle-related gene p21 and cell proliferation-associated genes cyclinD1 and c-myc^32^. Since LHX6 was found to promote normal palate development through mediating cell cycle^33^, it is hypothesized that LHX6 may overcome the resistance to EGFR-TKIs in NSCLC through mediating the expression of cell cycle-, apoptosis- and proliferation-associated genes.

**Figure 2:**
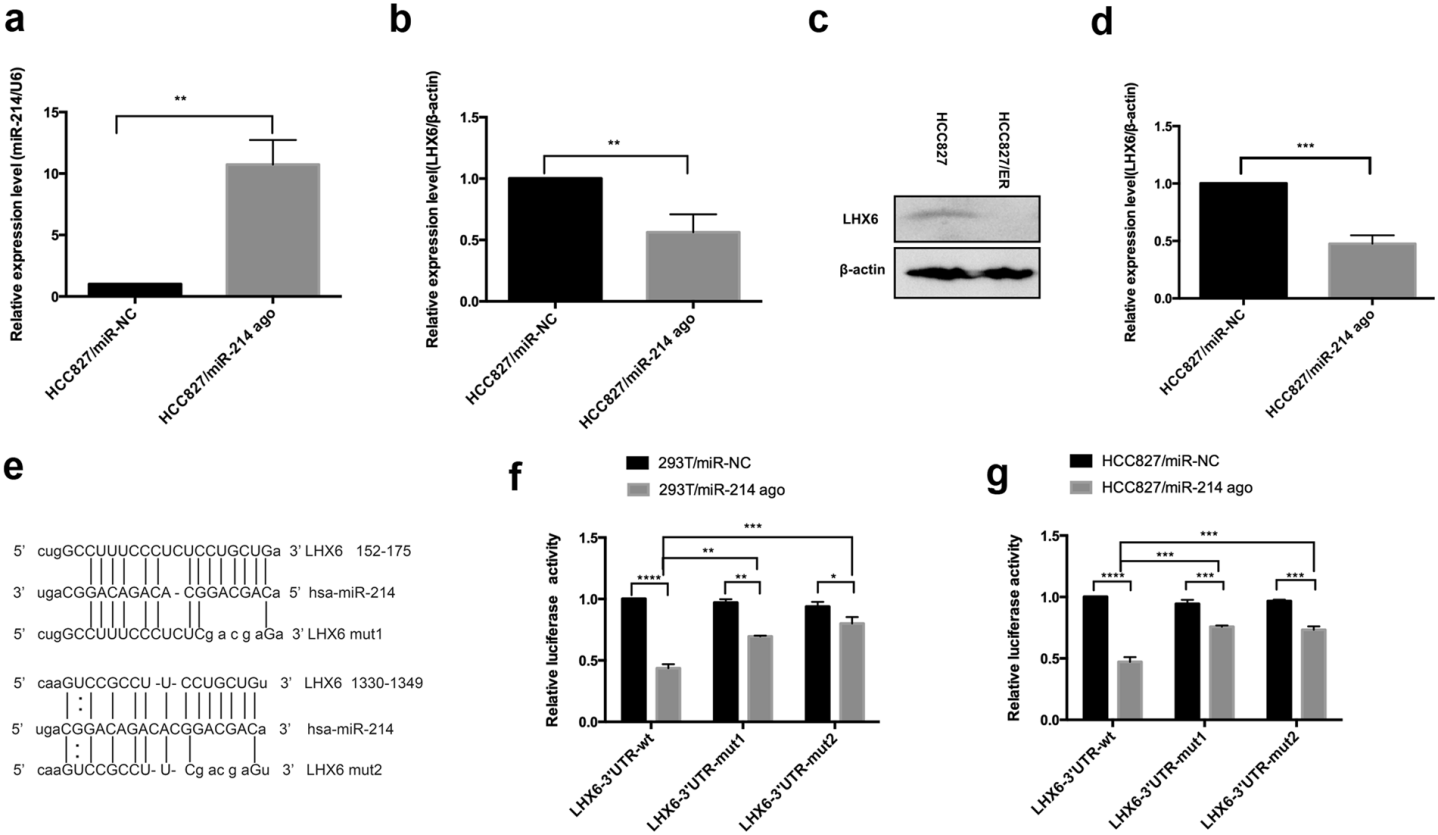
LHX6 is a direct target gene of miR-214. (**a**) Following 48 h transfection, qRT-PCR assay shows up-regulation of miR-214 expression in HCC827 cells transfected with miR-214 agomir than in cells transfected with miR-NC (*P* < 0.01); (**b**) qRT-PCR analysis reveals down-regulation of LHX6 expression in HCC827 cells transfected with miR-214 agomir than in cells transfected with miR-NC agomir (*P* < 0.01); (**c** and **d**) Western blotting assay reveals down-regulation of LHX6 protein expression in HCC827 cells transfected with miR-214 agomir than in cells transfected with miR-NC agomir (*P* < 0.0001); (**e**) Sequences of the 3′ UTR region of the LHX6 gene matching to miR-214. The 5′ terminus of miR-214, matched by the seed sequences of the LHX6 3′ UTR region, is ligated to the dual-luciferase reporter vector psi-check2 to construct wild-type (LHX6-3′ UTR/wt) and mutant (LHX6-3′ UTR/mut1 and LHX6-3′ UTR/mut2) dual-luciferase reporter genes; (**f** and **g**) Following co-transfection in 293 T (F) and HCC827 cells (G) with 50 nM miR-NC or miR-214 agomir in combination with 0.1 μg LHX6-3′ UTR-wt, LHX6-3′ UTR-mut1 or LHX6-3′ UTR-mut2 for 24 h, dual-luciferase reporter assay reveals a significant lower relative luciferase activity of LHX6-3′ UTR/wt in cells transfected with miR-214 agomir than in cells transfected with miR-NC, while significantly up-regulated relative luciferase activities of LHX6-3′ UTR/mut1 (*P* < 0.001) and LHX6-3′ UTR/mut2 (*P* < 0.001) were measured than that of LHX6-3′ UTR/wt in HCC827 and 293 T cells transfected with miR-214 agomir. All data are expressed as mean ± SD from three independent experiments. *P < 0.01; **P < 0.001; ***P < 0.01; ****P < 0.01.

To examine the link between miR-214 and LHX6, we predicted two miR-214 target sites on the 3′ UTR of LHX6 mRNA with bioinformatics tools. Following miR-214 agomir transfection, qRT-PCR analysis showed up-regulation of miR-214 expression in HCC827 cells (Fig. 2a), and down-regulation of LHX6 mRNA expression in HCC827 cells overexpressing miR-214 (Fig. 2b). In addition, Western blotting assay revealed a reduction in LHX6 protein expression in HCC827 cells harboring miR-214 overexpression (Fig. 2c and d).

The two miR-214 target sites were spliced using an overlapping extension PCR assay^34^, and then ligated to the dual-luciferase reporter vector psi-check2 to construct wild-type (LHX6-3′ UTR/wt) and mutant (LHX6-3′ UTR/mut1 and LHX6-3′ UTR/mut2) dual-luciferase reporter genes (Fig. 2e). The plasmid was then co-transfected with miR-NC or miR-214 agomir into HCC827 and 293 T cells. The relative luciferase activity of LHX6-3′ UTR/wt was significantly down-regulated in 293 T cells transfected with miR-214 agomir than in cells transfected with miR-NC agomir (*P* < 0.0001), while higher relative luciferase activities of LHX6-3′ UTR/mut1 (*P* < 0.01) and LHX6-3′ UTR/mut2 (*P* < 0.0001) were detected than that of LHX6-3′ UTR/wt in miR-214 agomir-transfected 293 T cells (Fig. 2f). There was a significant lower relative luciferase activity of LHX6-3′ UTR/wt in HCC827 cells transfected with miR-214 agomir than in cells transfected with miR-NC agomir (*P* < 0.0001), while significantly up-regulated relative luciferase activities of LHX6-3′ UTR/mut1 (*P* < 0.001) and LHX6-3′ UTR/mut2 (*P* < 0.001) were measured than that of LHX6-3′ UTR/wt in miR-214 agomir-transfected HCC827 cells (Fig. 2g). Our findings demonstrate that miR-214 may simultaneously regulate the two target sites of the LHX6 3′ UTR region to mediate LHX6 expression, indicating that LHX6 is a direct target gene of miR-214.

### LHX6 expression is down-regulated in HCC827/ER cells

**Figure 3:**
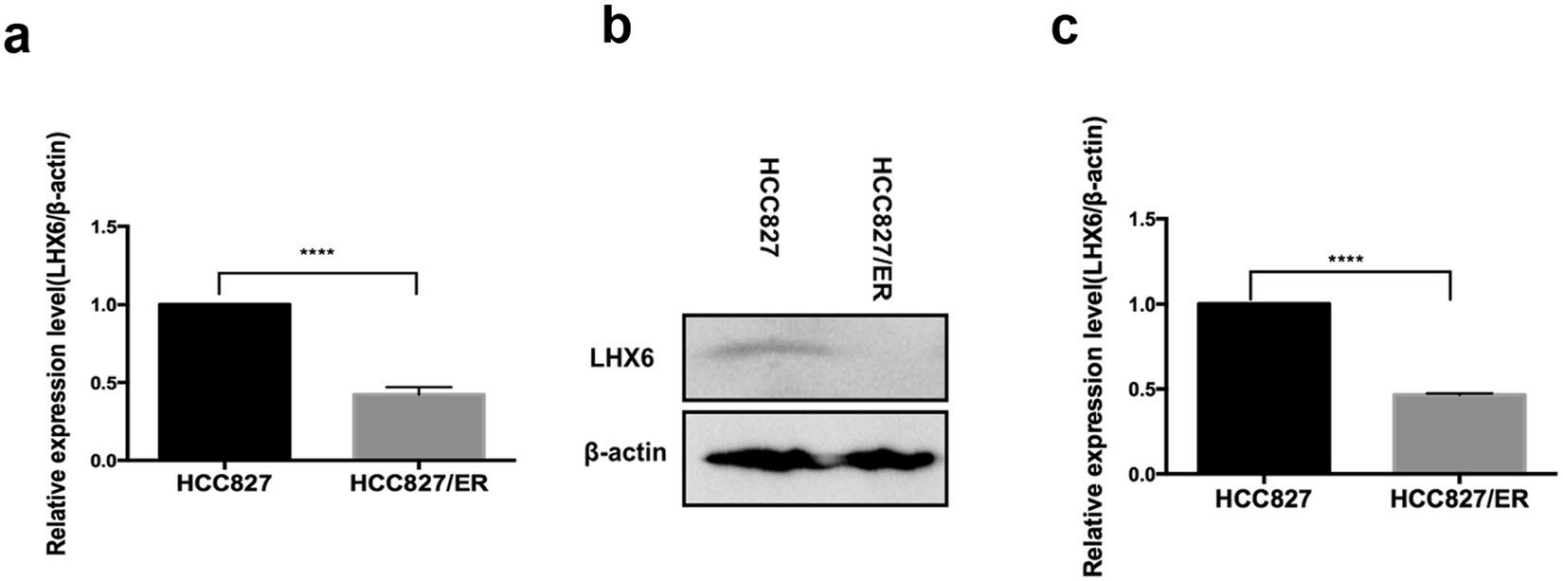
LHX6 expression is down-regulated in HCC827/ER cells at both translational and transcriptional levels. (**a**) qRT-PCR assay reveals down-regulation of LHX6 mRNA expression in HCC827/ER cells than in HCC827 cells (*P < *0.0001); (**b** and **c**) Western blotting analysis shows down-regulation of LHX6 protein expression in HCC827/ER cells than in HCC827 cells (*P* < 0.0001). All data are expressed as mean ± SD from three independent experiments. ****P < 0.01.

qRT-PCR and Western blotting assays showed down-regulation of LHX6 expression in HCC827/ER cells at both transcriptional and translational levels (Fig. 3). As shown above, miR-214 expression was found to be up-regulated in HCC827/ER cells and in the plasma of EGFR-mutant NSCLC patients with acquired EGFR-TKI resistance. Taking these results together, it is indicated that miR-214 expression correlates negatively with LHX6 expression. It is therefore hypothesized that LHX6 is involved in miR-214-mediated acquired resistance to EGFR-TKIs in EGFR-mutant NSCLC cell lines and patients.

### MiR-214 mediates resistance to EGFR-TKI through regulating LHX6 expression

**Figure 4:**
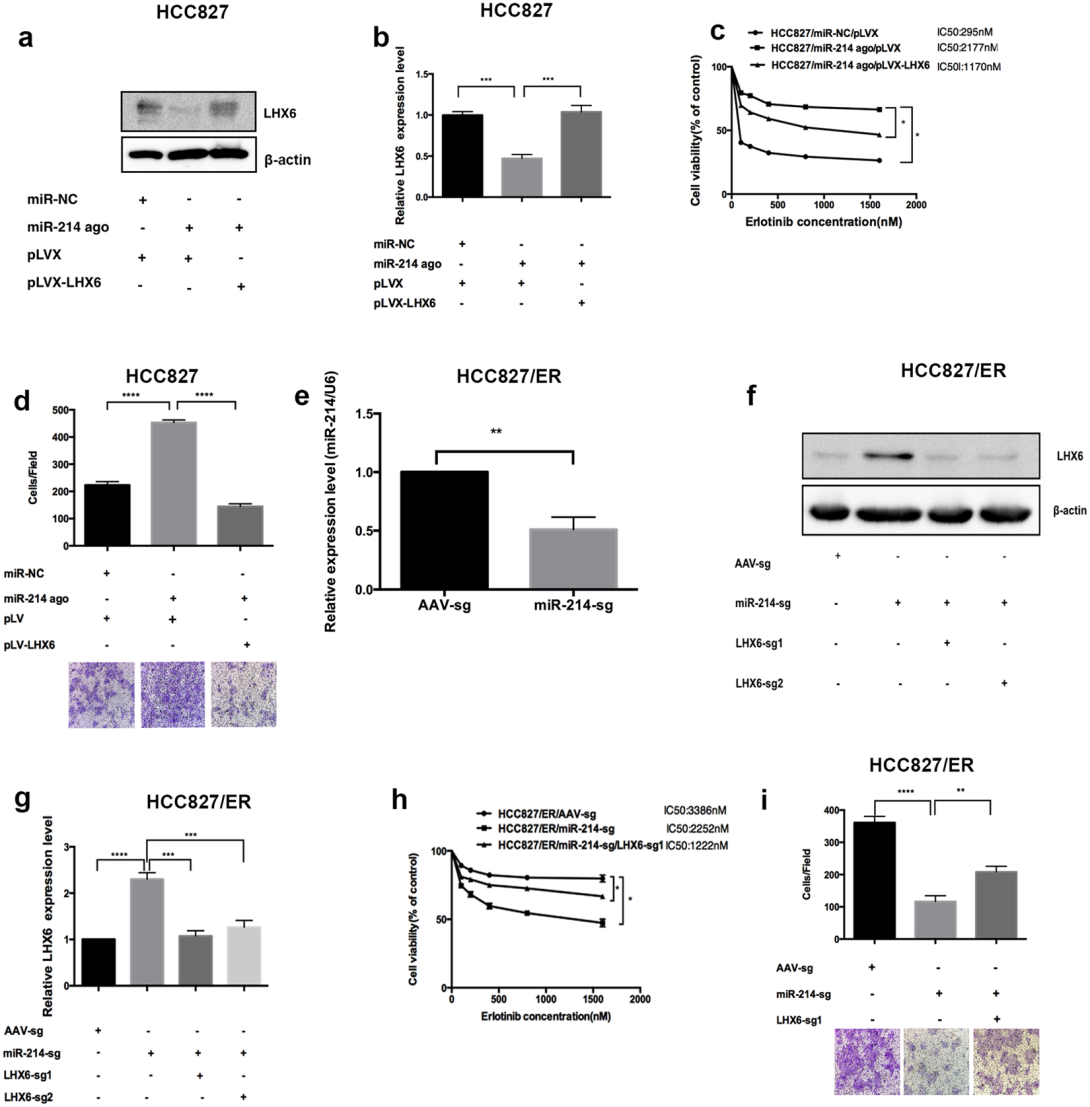
Effect of miR-214 expression on the sensitivity to EGFR-TKI in HCC827 and HCC827/ER cells. (**a** and **b**) Western blotting analysis determines LHX6 protein expression in HCC827 cells, HCC827 cells transfected with miR-214 agomir for 48 h, and HCC827 cells co-transfected with miR-214 agomir and pLV-LHX6 for 48 h, respectively; (**c**) MTS assay measures 72 h erlotinib IC_50_ values in HCC827 cells, HCC827 cells transfected with miR-214 agomir for 24 h, and HCC827 cells co-transfected with miR-214 agomir and pLV-LHX6 for 24 h, respectively; (**d**) Transwell invasion assay measures the counts of membrane-penetrating cells in HCC827 cells, HCC827 cells transfected with miR-214 agomir for 48 h, and HCC827 cells co-transfected with miR-214 agomir and pLV-LHX6 for 48 h, respectively; (**e**) Following miR-214 knockdown using the CRISPR/Cas9 system, qRT-PCR assay shows down-regulation of miR-214 expression in HCC827/ER/miR-214-CRI cells; (**f** and **g**) In the stably transfected HCC827/ER/mir-214-CRI cell line, sg1 and sg2 targeting LHX6 were designed and linked with the vector lentiCRISPRv2 to establish stably transfected HCC827/ER/miR-214-CRI/LHX6-sg1 and HCC827/ER/miR-214-CRI/LHX6-sg2 cell lines with miR-214 knockdown, and then, LHX6 protein expression is determined in these cell lines using Western blotting assay; (**h**) MTS assay reveals 72 h erlotinib IC_50_ values in these cell lines; (**i**) Transwell invasion assay measures the counts of membrane-penetrating cells. All data are expressed as mean ± SD from three independent experiments.

Western blotting analysis showed down-regulation of LHX6 protein expression in HCC827 cells transfected with miR-214 agomir (Fig. 4a and b). We then overexpressed LHX6 in HCC827 cells transfected with miR-214 agomir. MTS assay showed a 2,177 nM erlotinib IC_50_ for HCC827 cells co-transfected with miR-214 agomir and pLVX, a 295 nM erlotinib IC_50_ for HCC827 cells co-transfected with miR-NC agomir and pLVX, and a 1,170 nM erlotinib IC_50_ for HCC827 cells co-transfected with miR-214 agomir and LHX6 (Fig. 4c). The results indicated that LHX6 overexpression may reverse the reduced susceptibility to erlotinib in HCC827 cells, which was caused by miR-214 overexpression. Transwell invasion assay revealed that LHX6 overexpression reversed the increase in the invasive ability of HCC827 cells induced by overexpression of miR-214 (Fig. 4d).

MiR-214 expression was down-regulated in HCC827/ER cells by using the CRISPR/Cas9 system, and the stably transfected cell line HCC827/ER/mir-214-sg was established. qRT-PCR assay showed miR-214 down-regulation in HCC827/ER/mir-214-sg cells as compared to that in HCC827/ER cells transfected with AAV-sg (Fig. 4e). Following LHX6 knockdown in HCC827/ER/miR-214-sg cells using the CRISPR/Cas9 system, Western blotting assay revealed LHX6 down-regulation in LHX6-sg1 and LHX6-sg2 cells in relative to HCC827/miR-214-sg cells (Fig. 4f and g). MTS assay showed a 3,386 nM erlotinib IC_50_ for HCC827/ER/AAV-sg cells, 1,222 nM erlotinib IC_50_ for HCC827/ER/miR-214-CRI cells, and a 2,252 nM erlotinib IC_50_ for HCC827/ER/mir-214-CRI/LHX6-sg cells (Fig. 4h). Transwell invasion assay revealed that LHX6 knockdown reversed the reduction in the invasive ability of HCC827/ER cells induced by down-regulation of miR-214 (Fig. 4i).

## Discussion

To date, there have been many attempts to investigate the mechanisms underlying the acquired resistance to EGFR-TKIs in NSCLC, and secondary mutations, alternative signaling pathways, and phenotype changes have been linked to the emergence of acquired EGFR-TKI resistance in lung cancer^35–40^. However, the exact mechanisms underlying the acquired resistance to EGFR-TKI have not been fully demonstrated in NSCLC until now.

Gene post-transcriptional regulation has been found to confer the acquired resistance to EGFR-TKIs in NSCLC^41^, and miRNAs, as posttranscriptional gene regulators, are linked to chemotherapeutic drug resistance in multiple cancers^42, 43^. Previous studies have demonstrated the association of miR-214 with the resistance to chemotherapeutic agents in several types of cancers^27, 28^. A recent study showed the potential of miR-214 to overcome the resistance to tamoxifen and fulvestrant in estrogen receptor-positive breast cancer cells^44^, and miR-214 was reported to induce the resistance to cisplatin in human ovarian cancer cells via the PTEN/Akt pathway^29^. In addition, miR-214 down-regulation was found to confer the resistance to both P-glycoprotein-related and P-glycoprotein-nonrelated drugs in esophageal squamous cell carcinoma^45^, and this miRNA has been identified as a contributor to the resistance to cisplatin in tongue squamous cell carcinoma lines^46^. Although a link was detected between miR-214 and the acquired gefitinib resistance in EGFR-mutant NSCLC cell lines via the PTEN/AKT signaling pathway^30^, the role of miR-214 in the acquired resistance to EGFR-TKIs and the underlying mechanisms have not been fully understood in NSCLC.

In this study, we detected an increase in miR-214 expression in the plasma of NSCLC patients with acquired EGFR-TKI resistance than prior of EGFR-TKI therapy (*P* = 0.398). To examine the exact role of miR-214 in the acquired resistance to EGFR-TKI, we generated an erlotinib-resistant HCC827/ER cell line by drug selection at dose escalation. qRT-PCR assay detected up-regulation of miR-214 expression in HCC827/ER cells than in HCC827 cells, indicating that miR-214 may be involved in the acquired erlotinib resistance in NSCLC cell lines. Our findings are consistent with the previous study reporting higher miR-214 expression in gefitinib-resistant HCC827/GR cell lines than in HCC827 cells^30^. The findings suggest that elevated miR-214 expression may contribute to the acquired resistance to EGFR-TKI in NSCLC. Further studies recruiting more study subjects and NSCLC patients with EGFR 19del mutation are required to confirm the role of miR-214 in the acquired resistance to EGFR-TKIs.

LHX genes, one of the most important subfamilies of the homeobox genes, have been involved in multiple cancers^47^. For example, LHX1, LHX2 and LHX4 have been identified as oncogenes of nephroblastoma, clear cell renal cell carcinoma, chronic myeloid leukemia, and acute lymphoblastic leukemia, and LHX3, LHX5 and LHX6 were considered methylation biomarkers of breast cancer, head and neck squamous cell carcinoma and cervical cancer, while LHX9 and LMX1 A were recognized as tumor-suppressor genes of pediatric malignant astrocytomas, follicular lymphoma, cervical cancer, ovarian cancer and bladder cancer^47^. In addition, LHX6 was reported to suppress the growth and invasion of breast cancer cells through inhibiting Wnt/β-catenin signaling pathway^31^, and has been putatively identified as a tumor suppressor gene in lung cancer through regulating apoptosis-related genes p53 and Bcl-2, cell cycle-related gene p21 and cell proliferation-associated genes cyclinD1 and c-myc^32^. Since LHX6 was found to promote normal palate development through mediating cell cycle^33^, it is hypothesized that LHX6 may overcome the resistance to EGFR-TKIs in NSCLC through mediating the expression of cell cycle-, apoptosis- and proliferation-associated genes.

Bioinformatic analysis has predicted two target sites of miR-214 on the 3′ UTR of LHX6 mRNA^48^. In this study, miR-214 overexpression was found to cause suppression of LHX6 expression in HCC827 cells at both translational and transcriptional levels. The dual-luciferase assay reporter assay detected LHX6 as the direct target gene of miR-214, and down-regulation of LHX6 expression was determined in HCC827/ER cells. In addition, up-regulation of LHX6 expression was found to reverse the reduction in sensitivity to erotinib and the increase in the invasion and metastasis of HCC827 cells harboring miR-214 overexpression, and to reverse the increase in the sensitivity to erotinib and the decrease in the invasion and metastasis of HCC827/ER cells caused by down-regulation of miR-214. These findings indicate that miR-214 mediates the acquired resistance to erlotinib and invasive and metastatic ability of HCC827 cells via LHX6. Further studies are required to investigate the exact mechanisms underlying the involvement of LHX6 in miR-214-mediated acquired resistance to EGFR-TKIs in NSCLC.

Currently, miRNA expression is commonly inhibited by using chemically modified antisense oligonucleotides; however, such a technique suffers from high cost and off-target effects^49–51^. In the present study, we knocked out miR-214 using the CRISPR/Cas9 system in HCC827/ER cells, and RT-PCR assay detected down-regulation of miR-214 expression; however, complete knock-out of miR-214 was not observed. The results are consistent with the previous report that small guide RNA (sgRNA) targeting the stem loop of short hairpin RNA (shRNA) partially but not completely inhibited RNA expression^52^.

In summary, the results of the present study demonstrate that miR-214 plays a critical role in the acquired resistance to erotinib in both NSCLC cells and NSCLC patients harboring EGFR mutation, and knock-down of miR-214 may reverse erlotinib resistance in NSCLC through mediating its direct target gene LHX6 expression.

## Materials and Methods

### Study subjects

A total of 7 EGFR-mutant NSCLC patients that underwent EGFR-TKI therapy in Fujian Provincial Cancer Hospital (Fuzhou, China) during the period from June 2013 through March 2016 were enrolled in this study. The subjects included 2 men and 5 women, and 6 nonsmokers and 1 current smokers. Pathologic examinations revealed adenocarcinoma in all patients, and the staging of NSCLC was evaluated with the Seventh Edition of the American Joint Committee on Cancer (AJCC) Cancer Staging Manual^36^ (Table 1). The patients were given gefitinib (AstraZeneca Pharmaceuticals; Waltham, MA, USA) at a daily dose of 250 mg and erlotinib at a daily dose of 150 mg until tumor progression, and the objective response to treatment was assessed with the Response Evaluation Criteria in Solid Tumors (RECIST) version 1.1^37^. Route laboratorial testing and chest X-ray scan were performed once every two weeks, and CT scanning was performed at gefibinib administration, one month post-treatment with gefibinib, and once every three months post-treatment with gefibinib.

**Table 1:** Demographic and clinical characteristics of the study subject.

Case No.	Age (years)	Gender	Staging	Histopathology	PFS of TKI treatment (month)	TKI regimen	Mutation type
1	44	M	IV	Adenocarcinoma	29	Second-line	Exon *21 L858R*
2	63	F	IV	Adenocarcinoma	1	Second-line	Exon *21 L858R*
3	56	F	IV	Adenocarcinoma	17	Second-line	Exon *21 L858R*
4	60	F	III	Adenocarcinoma	8	First-line	Exon *21 L858R*
5	66	M	IV	Adenocarcinoma	20	First-line	Exon *21 L858R*
6	62	F	IV	Adenocarcinoma	11	First-line	Exon *21 L858R*
7	61	F	IV	Adenocarcinoma	1	Second-line	Exon *21 L858R*

### Cell culture and induction of erlotinib resistance in NSCLC cell lines

NSCLC HCC827 cell line expressing EGFR exon 19 deletion mutation was purchased from American Type Culture Collection (Manassas, VA, USA) and incubated in RPMI 1640 medium (Gibco; Garlsbad, CA, USA) supplemented with 10% fetal bovine serum (FBS; Gibco; Garlsbad, CA, USA), 100 U/ml penicillin and 100 µg/ml streptomycin at 37 °C in a humidified atmosphere containing 5% CO_2_. HCC827 cells were subjected to erlotinib (Selleckchem; Houston, TX, USA) treatment by dose escalation from 10 to 1,600 nM in RPMI 1640 medium containing 10% FBS. Following 6-month drug selection, HCC827 cells were transferred to erlotinib-free RPMI 1640 medium for a further incubation for 2 months (HCC827/ER cells).

HCC827 and HCC827/ER cells were seeded onto 96-well plates (Nalge Nunc International Corporation; Rochester, NY, USA) at a density of 5,000 cells per well. After 24 h, RPMI 1640 media supplemented with 10% FBS, which contained 100, 200, 400, 800 and 1,600 erlotinib were transferred for 72 h culture, while erlotinib-free RPMI 1640 medium served as controls. The viability of HCC827 and HCC827/ER cells was measured with the MTS assay (Promega; Madison, WI, USA). All experiments were repeated in triplicate. Finally, the experimentally induced HCC827/ER cells were identified erlotinib resistant.

### qRT-PCR assay

Approximately 10 ml of blood samples were centrifuged at 2,000 g at 4 °C for 10 min, and the supernatant was collected and centrifuged at 14,000 g at 4 °C for 10 min. The supernatant was collected and used for the subsequent experiments. miRNA was extracted from 200 µl of plasma samples using the miRcute miRNA isolation kit (Tiangen Biotech (Beijing) Co., Ltd.; Beijing, China), dissolved in 30 µl of RNAase-free water, and then stored at −80 °C for the subsequent experiments. Plasma miRNA and total RNA were reversely transcribed into cDNA with the miScript II RT Kit (Qiagen, Inc.; Valencia, CA, USA), and the *miR-214* expression was quantified with the miScript SYBR Green PCR Kit (Qiagen, Inc.; Valencia, CA, USA) on a LightCycler^®^ 480 II System (Roche Applied Science; Indianapolis, IN, USA) under the following conditions: at 95 °C for 15 min, followed by 40 cycles of at 94 °C for 15 s, at 55 °C for 30 s and at 70 °C for 30 s, while cel-miR-39 (5′-TCACCGGGTGTAAATCAGCTTG-3′) served as an internal reference.

HCC827 and HCC827/ER cells were digested in 0.25% Trypsin-EDTA (Gibco; Rockville, MD, USA), and then washed twice in PBS. Total RNA was extracted using a Trizol kit (Thermo Fisher Scientific; Waltham, MA, USA), and RNA concentration was quantified with the Nanodrop^®^ ND-1000 Spectrophotometer (Thermo Fisher Scientific; Waltham, MA, USA). Approximately 1 µg of total RNA sample was transcribed reversely into cDNA using the RevertAid First Strand cDNA Sythesis Kit (Thermo Fisher Scientific; Waltham, MA, USA) and LHX6 expression was determined with the Lightcycler 480 SYBR Green I Master (Roche Applied Science; Indianapolis, IN, USA) under the following conditions: at 95 °C for 15 min, followed by 40 cycles of at 95 °C for 15 s, at 55 °C for 30 s and at 72 °C for 1 s, while β-actin served as an internal control (primers are shown in Table 2).

**Table 2:** Primers and oligos used for qRT-PCR assay, psi-check2 and lentiCRISPR v2 cloning.

Primer	Sequence	Length (bp)
LHX6-F	5′-CGGAACAGCTGCAGGTTATG-3′	20
LHX6-R	5′-CTGAACGGGGTGTAGTGGAT-3′	20
*β-*actin-F	5′-CATCCGCAAAGACCTGTACG-3′	20
*β-*actin-R	5′-CATCCGCAAAGACCTGTACG-3′	20
LHX6-3′ UTR-Mut-F1	5′-TTTCCCTCTCGGACGAGAGAACCAGAAC-CCACCAGGAGCACC-3′	41
LHX6-3′ UTR-Mut-R1	5′-GGCCAGGCCTCGGGAACC-3′	18
LHX6-3′ UTR-Mut-F2	5′-AGTCCCCTTCgacgaGTGGTTAACCTGTTA-TGTTG-3′	35
LHX6-3′ UTR-Mut-R2	5′-TGGGCTAAAACATGCTGATTAG-3′	22
LHX6-sg1-F	5′-CACCGCGTTCTCGGCGGCCCTCTTG-3′	25
LHX6-sg1-R	5′-AAACCAAGAGGGCCGCCGAGAACGC-3′	25
LHX6-sg2-F	5′-CACCGGAAGGACGTCCGCGCGCGCT-3′	25
LHX6-sg2-R	5′-AAACAGCGCGCGCGGACGTCCTTCC-3′	25
miR-214-sg-F	5′-CACCGCATCCGCTCACCTGTACAGC-3′	25
miR-214-sg-R	5′-AAACGCTGTACAGGTGAGCGGATGC-3′	25
AAV-sg-F	5′-CACCGGGGCCACTAGGGACAGGAT-3′	24
AAV-sg-R	5′-AAACATCCTGTCCCTAGTGGCCCC-3′	24

Relative quantity of miR-214 and LHX6 mRNA expression was calculated by using the 2^−ΔΔCT^ method. All measurements were repeated in triplicate.

### Western blotting analysis

HCC827 and HCC827/ER cells were digested in 0.25% Trypsin-EDTA, washed twice in PBS, and completely lysed in cell lysis buffer (Cell Signaling Technology; Beverly, MA, USA) on ice. The solution was centrifuged at 12,000 g at 4 °C for 10 min and the supernatant was collected. The concentration of total protein was quantified with the BCA Protein Assay Kit (Thermo Fisher Scientific; Waltham, MA, USA) and total protein was separated with SDS-PAGE. Subsequently, the blots were transferred to nitrocellulose (NC) membranes and blocked in 3% bovine serum albumin (BSA) at 25 °C for 1 h. Then, the blots were incubated in mouse monoclonal anti-LHX6 antibody (1:500 dilution; Santa Cruz Biotechnology; Santa Cruz, CA, USA) at 4 °C overnight, while β-actin was used as a loading control. The blots were then washed three times in TBST (20 mM Tris-HCl, 150 mM NaCl and 0.05% Tween-20; pH 7.4), of 10 min each time, incubated in anti-rabbit/mouse HRP-conjugated IgG antibody (1:4,000 dilution; Cell Signaling Technology; Beverly, MA, USA) at 25 °C for 3 h, and washed three times in TBST, of 10 min each time. The protein bands were visualized using an ECL Kit (Thermo Fisher Scientific; Waltham, MA, USA), and the expression level of each protein was normalized to that of β-action. All measurements were repeated in triplicate.

### Transwell invasion assay

HCC827 cells were seeded onto 6-well plates at a density of 5 × 10^3^ cells per well. After 24 h, 50 nM miR-NC or miR-214 agomir, and pLVX or pLVX-LHX6 were transferred for 48 h incubation. Then, cells were harvested and transferred to serum-free medium, with the cell density adjusted to 4 × 10^5^ cells/ml. The stably transfected cell line HCC827/ER/AAV-sg, which was obtained by infection of HCC827/ER cells with AAV-sg vector, and the stably transfected cell line HCC827/ER/miR-214-sg, which was obtained by infection of HCC827/ER cells with miR-214-sg vector, were infected with LHX6-sg1 vector to construct HCC827/ER/miR-214-sg/LHX6-sg1 cell line in which both miR-214 and LHX6 were knocked out, and the cell density was adjusted to 4 × 10^5^ cells/ml. Approximately 100 µl of cells were transferred to the upper chamber, and the bottom of the upper chamber was coated with 1 mg/ml fibronetin (Millipore; Bedford, CA, USA), while the lower chamber was transferred with the medium containing 20% FBS. Following incubation at 37 °C for 48 h, cells were gently removed from the upper chamber using cotton swabs, and the chamber was fixed in methanol for 15 min, dried and stained with 0.1% crystal violet. Five fields of vision were randomly selected, and the number of cells in each field was counted under an inverted microscope at a magnification of ×200. All experiments were repeated in triplicate.

### Dual-luciferase reporter assay

HCC827 and 293 T cells were seeded onto 96-well plates at a density of 5 × 10^3^ cells per well. Following 24 h incubation, the supernatant was removed and 50 nM miR-NC and miR-214 agomir was transferred. The two miR-214 target sites on the 3′ UTR of LHX6 mRNA were spliced with an overlapping extension PCR with Ex-Taq (Takara; Dalian, China), cloned into pGEM-T Easy vector (Promega; Madison, WI, USA), digested with Xho I and Not I, and ligated to the psi-check2 vector (Promega; Madison, WI, USA). The mutant vectors psi-check2-LHX6 mut1 and psi-check2-LHX6 mut2, which targeted the two target sites on the 3′ UTR of LHX6 mRNA, were constructed with the Q5 Site-Directed Mutagenesis Kit (New England Biolabs; Ispswich, MA, USA) following the manufacturer’s instructions. Then, psi-check2-LHX6 wt, psi-check2-LHX6 mut1 or psi-check2-LHX6 mut2 was co-transfected with miR-NC or miR-214 agomir into 293 T and HCC827 cells with the X-treme GENE HP DNA Transfection Reagent (Roche NimbleGen; Madison, WI, USA) according to the manufacturer’s protocol. Following 48 h transfection, the Fly and Renilla luciferase activities were measured with the Dual-Glo luciferase assay system on a Synergy H4 Hybrid Multi-Mode Microplate Reader (BioTeK; Winooski, VT, USA). All measurements were repeated in triplicate.

### Vector construction and viral infection in cells

The start codon of LHX6 was selected as the target of sgRNA, and two pairs of oligonucleotides were designed for sgRNA using the online CRISPR software (http://crispr.mit.edu/), while AAV-sg served as a control. In addition, the sgRNA oligonucleotide miR-214-sg that targeted the stem-loop region of miR-214 was designed with the CRISPR tool. The oligonucleotide pairs were annealed, ligated to lentiCRISPRv2, and transfected into Trans1-T1 Phage Resistant Chemically Competent Cells (Transgen Biotech; Beijing, China). The clones were selected and subjected to Sanger sequencing. The required plasmid was collected for the subsequent experiment. The expression vector lentiCRISPRv2-LHX6-sg1, lentiCRISPRv2-AAV-sg, lentiCRISPRv2-LHX6-sg1, lentiCRISPRv2-LHX6-sg2 or lentiCRISPRv2-miR214-sg was co-transfected with the packaging plus envelope plasmid pPAX2 and the packaging plasmid pCMV-VSVG into 293 T cells. At 24 and 48 h post-transfection, the cell culture supernatant was collected, centrifuged at 21,600 r/min at 4 °C for 2 h. Virus was filtered through the 0.22 µm PVDF membrane. HCC827/ER cells were infected with lentiCRISPRv2-AAV-sg, lentiCRISPRv2-LHX6-sg1, lentiCRISPRv2-LHX6-sg2 or lentiCRISPRv2-miR214, and added with 6 µg/ml polybrene. After 48 h infection, HCC827/ER cells were screened with 2 µg/ml puromycin for 2 weeks to generate stably transfected HCC827/ER/miR-214-sg, HCC827/ER/miR-214-sg/LHX6-sg1 and HCC827/ER/miR-214-sg/LHX6-sg2 cell lines.

### Ethical statement

This study was approved by the Ethical Review Committee of Fujian Provincial Cancer Hospital (approval no. FJZLYY2015-00179). Signed informed consent was obtained from all participants following a detailed description of the purpose of the study, and all subjects involved in this study agreed to publish related demographic and clinical features. All experimental procedures described in this study were conducted in accordance with national and local laws, regulations and guidelines.

### Data analysis

All data were expressed as mean ± standard deviation (SD), and all statistical analyses were performed using the software GraphPad Prism version 6.0. Differences of means between groups were tested for statistical significance with Student *t* test, and a *P* value < 0.05 was considered statistically significant.
